# Endurance exercise remodels skeletal muscle by suppressing Ythdf1-mediated myostatin expression

**DOI:** 10.1038/s41419-025-07379-5

**Published:** 2025-02-13

**Authors:** Xin Huang, Chenzhong Xu, Jie Zhang, Weiwei Wu, Zimei Wang, Qiuxiang Pang, Zuojun Liu, Baohua Liu

**Affiliations:** 1 Shenzhen Key Laboratory for Systemic Aging and Intervention (SKL-SAI), National Engineering Research Center for Biotechnology (Shenzhen), International Cancer Center, School of Basic Medical Sciences, Shenzhen University Medical School, Shenzhen, China; 2 Anti-aging & Regenerative Medicine Research Institution, School of Life Sciences, Shandong University of Technology, Zibo, China; 3 Hainan Province Key Laboratory of One Health, Collaborative Innovation Center of One Health, School of Life and Health Sciences, Hainan University, Haikou, Hainan China; 4 School of Environmental Science and Engineering, Hainan University, Haikou, Hainan China

**Keywords:** Muscle stem cells, RNA modification

## Abstract

Exercise can improve health via skeletal muscle remodeling. Elucidating the underlying mechanism may lead to new therapeutics for aging-related loss of skeletal muscle mass. Here, we show that endurance exercise suppresses expression of YT521-B homology domain family (Ythdf1) in skeletal muscle, which recognizes the N6-methyladenosine (m6A). *Ythdf1* deletion phenocopies endurance exercise-induced muscle hypertrophy in mice increases muscle mitochondria content and type I fiber specification. At the molecular level, Ythdf1 recognizes and promotes the translation of m6A-modified *Mstn* mRNA, which encodes a muscle growth inhibitor, Myostatin. Loss of *Ythdf1* leads to hyperactivation of skeletal muscle stem cells (MuSCs), also called satellite cells (SCs), enhancing muscle growth and injury-induced regeneration. Our data reveal Ythdf1 as a key regulator of skeletal muscle homeostasis, provide insights into the mechanism by which endurance exercise promotes skeletal muscle remodeling and highlight potential strategies to prevent aging-related muscle degeneration.

## Introduction

Skeletal muscle retains lifelong regeneration capacity owing to a designated population of muscle stem cells (MuSCs), also known as satellite cells (SCs) [1–3]. SCs are typically quiescent and characterized by the expression of transcription factor paired box 7 (Pax7) [4]. Upon muscle injuries, the quiescent SCs are evoked and differentiated, which is regulated by myogenic determination factor 1 (MyoD) [2, 3]. Various growth factors and cytokines regulate the growth and regeneration of skeletal muscles [5]. Of these, myostatin (Mstn), a TGF-β superfamily member and known as growth differentiation factor 8 (GDF8), acts as a negative regulator to prevent muscle overgrowth [6–8]. Mstn inhibits proliferation and differentiation of myoblasts via suppressing the expression of myogenic factors *MyoD* and *Myogenin* [9–12]. Depletion of *Mstn* in mice activates SCs and improves skeletal muscle regeneration [13, 14]. Mstn induces proteasomal degradation of proteins in catabolic muscle-wasting conditions [15–17], and inhibits protein translation through suppressing the IGF-1/PI3K/Akt/mTOR pathway [18, 19]. Interestingly, pharmacological blockade of Mstn signaling induces skeletal muscle hypertrophy without exhausting SCs pool [20, 21]. Thus, the Mstn signaling represents a potential target for the treatment of muscle atrophy [22].

Exercise training has notable benefits on human health, which are partially mediated by the remodeling of skeletal muscle [23, 24]. In response to exercise, SCs are activated, which is accompanied by an increase in expression of myogenic regulators [25, 26]. Accumulating evidence supports that RNA modifications act as an additional layer of regulation in skeletal muscle homeostasis. N6-methyladenosine (m6A) is one of the most abundant internal mRNA modifications. The YT521-B homology domain family (YTHDFs) and domain-containing (YTHDCs) proteins recognize m6A modification on RNAs to regulate their stability, splicing, translation, and translocation [27, 28]. The methyltransferase-like 3 (Mettl3) mediates mRNA m6A modification of activin type 2 A receptor (*Acvr2a*), an Mstn receptor; the m6A modification is recognized by Ythdf2 to induce its degradation [29]. Ythdf2 also enhances the degradation of ubiquitin ligase *Asb2* mRNA, thus synergistically inhibiting TGF-β/Smad3 signaling [30]. However, whether RNA m6A modification mediates endurance exercise-induced skeletal muscle remodeling is unknown.

In this study, we generated a murine *Ythdf1* knockout (KO) allele and found that *Ythdf1* deletion hyperactivated SCs, induced skeletal muscle hypertrophy, and enhanced endurance exercise capacity. At the molecular level, Ythdf1 recognizes m6A-modified *Mstn* mRNA to promote its translation. Our data reveal a positive regulator of the Mstn signaling thus ensuring the skeletal muscle homeostasis.

## Results

### Endurance exercise inhibits m6A and *Ythdf1* in skeletal muscle

**Fig. 1:**
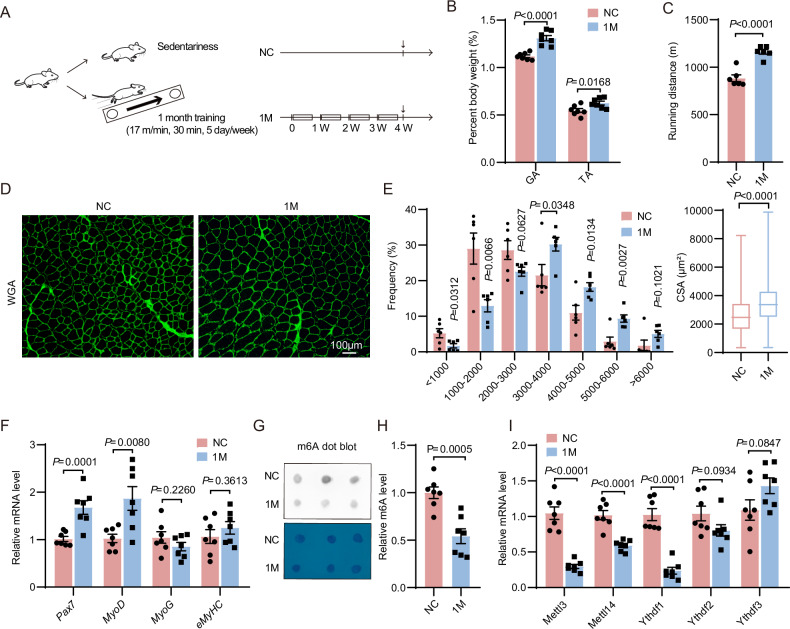
m6A and *Ythdf1* are regulated by exercise training. **A** Schematic of endurance exercise training of mice. NC, sedentary control; M, month; W, week. **B** The skeletal muscle mass in sedentary or trained mice (*n* = 7). GA, gastrocnemius; TA, tibialis anterior. **C** Running distance of sedentary or trained mice (*n* = 6). **D** WGA staining of gastrocnemius muscles from sedentary or trained mice. Scale bar, 100 µm. **E** Quantification of cross-sectional area (CSA) of myocytes in (**D**) (*n* = 6). **F** qRT-PCR analysis of gene expression levels in gastrocnemius muscles of sedentary or trained mice (*n* = 7). **G** Representative dot-blots with anti-m6A antibody showing m6A levels on RNAs of gastrocnemius muscles in sedentary or trained mice. **H** Quantification of m6A levels in (**G**) (*n* = 7). **I** qRT-PCR analysis of mRNA levels in gastrocnemius muscles of sedentary or trained mice (*n* = 7). Data represent the means ± SEM. *P* values calculated by two-tailed unpaired Student’s *t*-test.

To investigate whether RNA m6A modification is involved in exercise-induced skeletal muscle remodeling, the mice were subjected to a moderate-intensity endurance exercise training (Fig. 1A). After 4 weeks of exercise, skeletal muscle mass, running distance, and area of myofibers were all significantly increased in the trained mice compared to the sedentary controls (Fig. 1B–E, *P* < 0.05), suggesting skeletal muscle remodeling. Meanwhile, mRNA levels of two key myogenic factors, *Pax7* and *MyoD*, were elevated by exercise training, suggesting activation of SCs (Fig. 1F, *P* < 0.01). Interestingly, dot-blotting showed that exercise training strongly inhibited the m6A levels of RNAs in skeletal muscle (Fig. 1G, H, *P* = 0.0005). Consistently, the expression of m6A methyltransferase complex, *Mettl3* and *Mettl14*, were both downregulated by exercise. In addition, the mRNA level of *Ythdf1* but not *Ythdf2/3* was significantly reduced after exercise (Fig. 1I, *P* < 0.0001). These data suggest that m6A and *Ythdf1* are associated with exercise-induced skeletal muscle remodeling.

### Loss of *Ythdf1* enhances aerobic capacity of skeletal muscle in mice

**Fig. 2:**
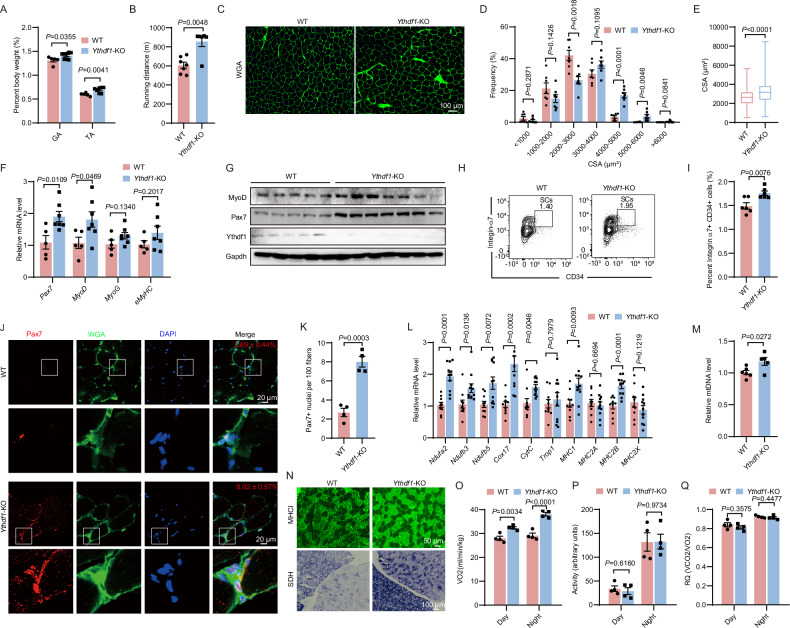
Loss of *Ythdf1* enhanced oxidative capacity and running endurance in mice. **A** Weight of gastrocnemius (GA) and tibialis anterior (TA) muscles in *Ythdf1*^−/−^ and WT mice aged 3 months (*n* = 5/8). **B** Treadmill running endurance test of *Ythdf1*^−/−^ and WT mice aged 3 months (*n* = 6/7). **C** Representative images of WGA staining of gastrocnemius muscles in *Ythdf1*^−/−^ and WT mice aged 3 months. Scale bar = 100 μm. **D**, **E** Quantification of muscle fiber size (cross-sectional area; CSA) and frequency in (**C**) (*n* = 7). **F** qPCR analysis of mRNA levels of the indicated genes in tibialis anterior muscles of *Ythdf1*^−/−^ and WT mice (*n* = 5/6). **G** Representative immunoblots showing Pax7, MyoD, and Ythdf1 protein levels in gastrocnemius muscles of *Ythdf1*^−/−^ and WT mice aged 3 months. Flow cytometry gating strategy (**H**) and percentage of SC population (CD34^+^integrin-α7^+^CD45^−^Sca1^−^CD11b^−^CD31^−^) (**I**) in gastrocnemius muscles from *Ythdf1*^−/−^ and WT mice aged 3 months (*n* = 6). **J** Immunofluorescent staining of Pax7 in gastrocnemius muscles from *Ythdf1*^−/−^ and WT mice. Scale bar = 20 μm. **K** Quantification of muscle Pax7^+^ cells in (**J**) (*n* = 4). **L** qPCR analysis of the indicated gene expression in gastrocnemius muscles of *Ythdf1*^−/−^ and WT mice aged 3 months (*n* = 10/11). **M** qPCR analysis of mitochondrial DNA in gastrocnemius muscles of *Ythdf1*^−/−^ and WT mice aged 3 months (*n* = 5/6). **N** MHC-I immunofluorescence staining (scale bar = 50 μm) and SDH staining (scale bar = 100 μm) in gastrocnemius muscles from *Ythdf1*^−/−^ and WT mice aged 3 months. O_2_ consumption (**O**), locomotor activity (**P**), and respiratory quotient (**Q**) of *Ythdf1*^−/−^ and WT mice aged 3 months determined by indirect calorimetry (*n* = 4). Data represent the means ± SEM. *P* values were calculated by two-tailed unpaired Student’s *t*-test.

To investigate whether Ythdf1 underlies exercise-induced skeletal muscle remodeling, we generated a murine *Ythdf1* knockout (KO) allele using the CRISPR/Cas9 system (Fig. S1A). Successful deletion of *Ythdf1* in various tissues of *Ythdf1*^−/−^ mice was confirmed by PCR-based genotyping as well as protein and mRNA level analyses (Fig. S1B–D). Compared with the wild-type (WT), the mass of gastrocnemius and tibialis anterior muscles were both significantly increased in *Ythdf1*^−/−^ mice (Fig. 2A, *P* < 0.05), and the running performance was significantly enhanced (Fig. 2B, *P* < 0.005). WGA staining showed that the cross-sectional area of myofibers in *Ythdf1*^−/−^ mice was significantly larger than that in the WT (Fig. 2C–E, *P* < 0.0001 in E). We next examined whether the increased muscle mass could be attributed to muscle protein synthesis and/or myogenic differentiation. Indeed, the mRNA levels of *Pax7* and *MyoD*, which mediate the activation of SCs from quiescence, were both significantly increased in the gastrocnemius muscles (Fig. 2F, *P* < 0.05). A concomitant increase in the protein levels of Pax7 and MyoD was observed in the gastrocnemius muscles of *Ythdf1*^−/−^ mice (Fig. 2G), suggesting an enhanced proliferative capacity of SCs. In contrast, there was little difference between the *Ythdf1*^−/−^ and WT mice in terms of expression levels of *MyoG* and *eMyHC*, which promote SC differentiation (Fig. 2F). We next examined SC numbers by FACS and immunofluorescence (IF) staining against Pax7. The number of SCs was significantly increased in the muscle from *Ythdf1*^−/−^ mice compared with that from WT (Fig. 2H–K, FACS: *P* = 0.0076; IF: *P* = 0.0003).

Enhanced running performance of *Ythdf1*^−/−^ mice suggests that the oxidative capacity of muscle might be affected. Therefore, we examined the expression of biomarkers of oxidative metabolism. Indeed, the mRNA levels of biomarkers of oxidative muscle fibers (*MHC-I*) and genes involved in mitochondrial respiration (*Ndufa2*, *Ndufb3*, *Ndufb5*, *Cox17*, and *CytC*) were all significantly upregulated in the gastrocnemius muscles of *Ythdf1*^−/−^ mice (Fig. 2L, *P* < 0.02). In addition, increased levels of mitochondrial DNA indicated a greater content of mitochondria (Fig. 2M, *P* = 0.0272). Furthermore, the numbers of MHC-I-positive and succinate dehydrogenase (SDH)-positive fibers were markedly increased in the gastrocnemius muscles of *Ythdf1*^−/−^ mice (Fig. 2N). Increased muscle oxidative metabolism is associated with higher energy expenditure. Indirect calorimetry indicated that *Ythdf1*^−/−^ mice consumed more O_2_ than the WT (Fig. 2O, *P* < 0.005), while there was little difference in the respiratory quotient and spontaneous activity between the two groups (Fig. 2P, Q). *Ythdf1* deletion may affect mitochondrial biogenesis in other highly metabolic tissues, such as brown adipose tissue. Interestingly, we were unable to detect any change in the levels of the metabolic genes after *Ythdf1* deletion (Fig. S1E), supporting its major impact on skeletal muscle. Together, these data indicate that *Ythdf1* deficiency induces skeletal muscle remodeling.

### Ythdf1 suppresses proliferation and differentiation of primary myoblasts

**Fig. 3:**
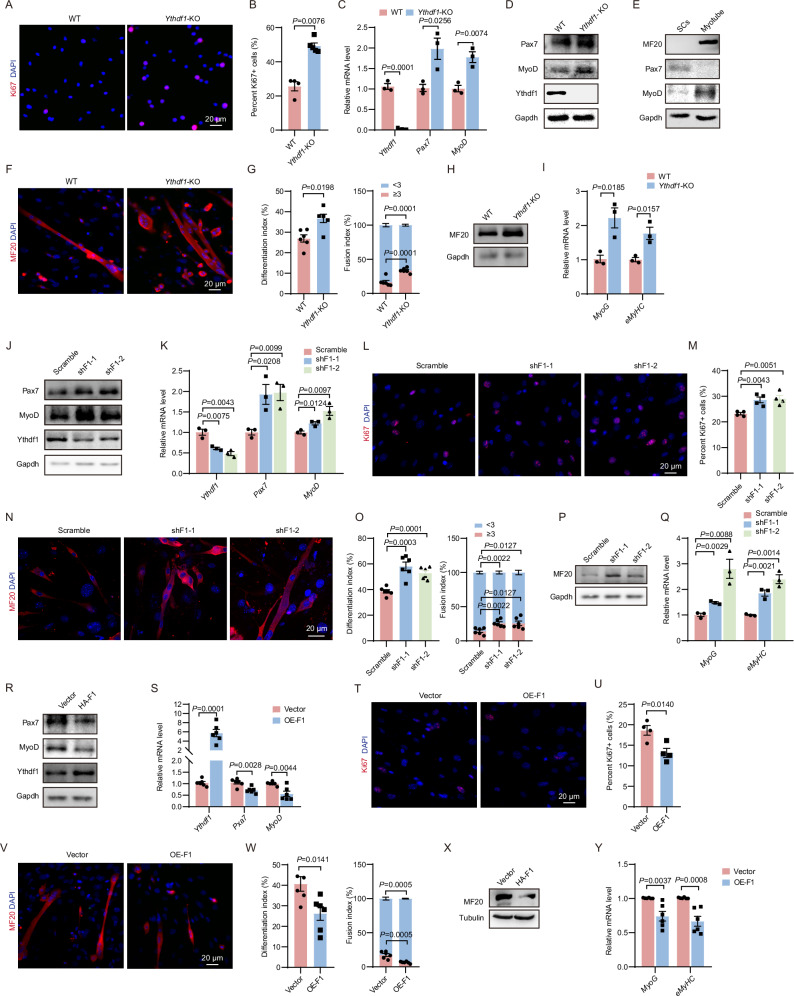
Ythdf1 regulates proliferation and differentiation of primary myoblasts. **A** Immunofluorescence staining of Ki67 in *Ythdf1*^*−/−*^ and WT primary myoblasts. Scale bar = 20 μm. **B** Quantification of Ki67^+^ cells in (**A**). **C** qRT-PCR analysis of mRNA levels of *Ythdf1*, *Pax7*, and *MyoD* in *Ythdf1*^−/−^ and WT primary myoblasts. **D** Representative immunoblots showing protein levels of Ythdf1, Pax7, and MyoD in *Ythdf1*^−/−^ and WT primary myoblasts. **E** Representative immunoblots showing protein levels of MF20, Pax7, and MyoD in differentiated myotubes and primary myoblasts. **F** Immunofluorescence staining of MF20 in differentiated myotubes from *Ythdf1*^−/−^ and WT primary myoblasts. Scale bar = 20 μm. **G** Quantification of differentiation index and fusion index in (**F**). **H** Representative immunoblots showing protein levels of MF20 in differentiated *Ythdf1*^−/−^ and WT myotubes. **I** qRT-PCR analysis mRNA levels of *MyoG* and e*MyHC* in differentiated *Ythdf1*^−/−^ and WT myotubes. **J** Representative immunoblots showing protein levels of Ythdf1, Pax7, and MyoD in *Ythdf1* knockdown (KD) and control primary myoblasts using two different shRNAs. **K** qRT-PCR analysis of *Ythdf1*, *Pax7*, and *MyoD* mRNA levels in *Ythdf1* KD and control primary myoblasts. **L** Immunofluorescence staining of Ki67^+^ in *Ythdf1* KD and control primary myoblasts. Scale bar = 20 μm. **M** Quantification of Ki67^+^ cells in (**L**). **N** Immunofluorescence staining of MF20 in differentiated *Ythdf1* KD and control myotubes. Scale bar = 20 μm. **O** Quantification of differentiation index and fusion index in (**N**). **P** Representative immunoblots showing protein levels of MF20 in differentiated *Ythdf1* KD and control myotubes. **Q** qRT-PCR analysis of mRNA levels of *MyoG* and *eMyHC* in differentiated *Ythdf1* KD and control myotubes. **R** Representative immunoblots showing protein levels of Ythdf1, Pax7, and MyoD in *Ythdf1* overexpressing (OE) and control primary myoblasts. **S** qRT-PCR analysis of *Ythdf1*, *Pax7*, and *MyoD* mRNA levels in *Ythdf1* OE and control primary myoblasts. **T** Immunofluorescence staining of Ki67^+^ in *Ythdf1* OE and control primary myoblasts. Scale bar = 20 μm. **U** Quantification of Ki67^+^ cells in (**T**). **V** Immunofluorescence staining of MF20 in differentiated *Ythdf1* OE and control myotubes. Scale bar = 20 μm. **W** Quantification of differentiation index and fusion index in (**V**). **X** Representative immunoblots showing protein levels of MF20 in differentiated *Ythdf1* OE and control myotubes. **Y** qRT-PCR analysis mRNA levels of *MyoG* and *eMyHC* in differentiated *Ythdf1* OE and control myotubes. Data represent the means ± SEM. *P* values were calculated by two-tailed unpaired Student’s *t*-test.

SCs regulate muscle homeostasis [31, 32]. To determine the role of Ythdf1 in SCs, we isolated primary myoblasts from *Ythdf1*^−/−^ and WT mice (Fig. S2). IF staining showed a higher percentage of Ki67^+^ (SC proliferation marker) in *Ythdf1*^−/−^ myoblasts compared to controls (Fig. 3A, B, *P* = 0.0076). Similarly, the mRNA and protein levels of Pax7 and MyoD were significantly upregulated in *Ythdf1*^−/−^ myoblasts (Fig. 3C, D, *P* < 0.05). Next, we induced SC differentiation by incubation with 2% horse serum for 48 h. Downregulation of Pax7 protein levels was noted, accompanied by the appearance of MF20 protein (myotube marker) (Fig. 3E). Loss of *Ythdf1* strongly promoted differentiation of myoblasts to myotubes (Fig. 3F). Fusion index analysis indicated that *Ythdf1* deficiency led to a significant increase in the number of multinucleated MF20^+^ myotubes (Fig. 3G, *P* = 0.0001). Western blotting analysis confirmed the increase in the MF20 protein level in *Ythdf1*^−/−^ myotubes compared with controls (Fig. 3H). The mRNA levels of the differentiation-related genes *MyoG* and *eMyHC* were also significantly upregulated in *Ythdf1*^−/−^ myotubes (Fig. 3I, *P* < 0.02). Thus, *Ythdf1* loss enhances the proliferation and differentiation of primary myoblasts.

We next explored whether the SC hyperactivation is cell-intrinsic and caused primarily by *Ythdf1* depletion. To that end, we knocked down *Ythdf1* in primary myoblasts. In accordance with the observation in *Ythdf1*^−/−^ SCs, *Ythdf1* knockdown (KD) upregulated both the protein and mRNA levels of Pax7 and MyoD (Fig. 3J, K, *P* < 0.03). *Ythdf1* KD also induced proliferation and differentiation of SCs, indicated by the increased Ki67 and MF20 staining, respectively (Fig. 3L–O, *P* < 0.02). The protein level of MF20 was also elevated upon *Ythdf1* KD (Fig. 3P). In addition, *MyoG* and *eMyHC* expression levels were significantly elevated in *Ythdf1* KD primary myoblasts **(**Fig. 3Q, *P* < 0.03**)**. In contrast, *Ythdf1* overexpression (OE) led to reduced expression levels of Pax7 and MyoD (Fig. 3R, S, *P* < 0.005), and inhibited myoblast proliferation (Fig. 2T, U, *P* < 0.02). Following differentiation, *Ythdf1* OE decreased the formation of MF20-positive myotubes (Fig. 3V–X, *P* < 0.02). Moreover, the mRNA levels of *MyoG* and *eMyHC* were significantly reduced by *Ythdf1* OE (Fig. 3Y, *P* < 0.005). These data indicated that Ythdf1 inhibits SC proliferation and differentiation.

### Ythdf1 enhances *Mstn* translation

**Fig. 4:**
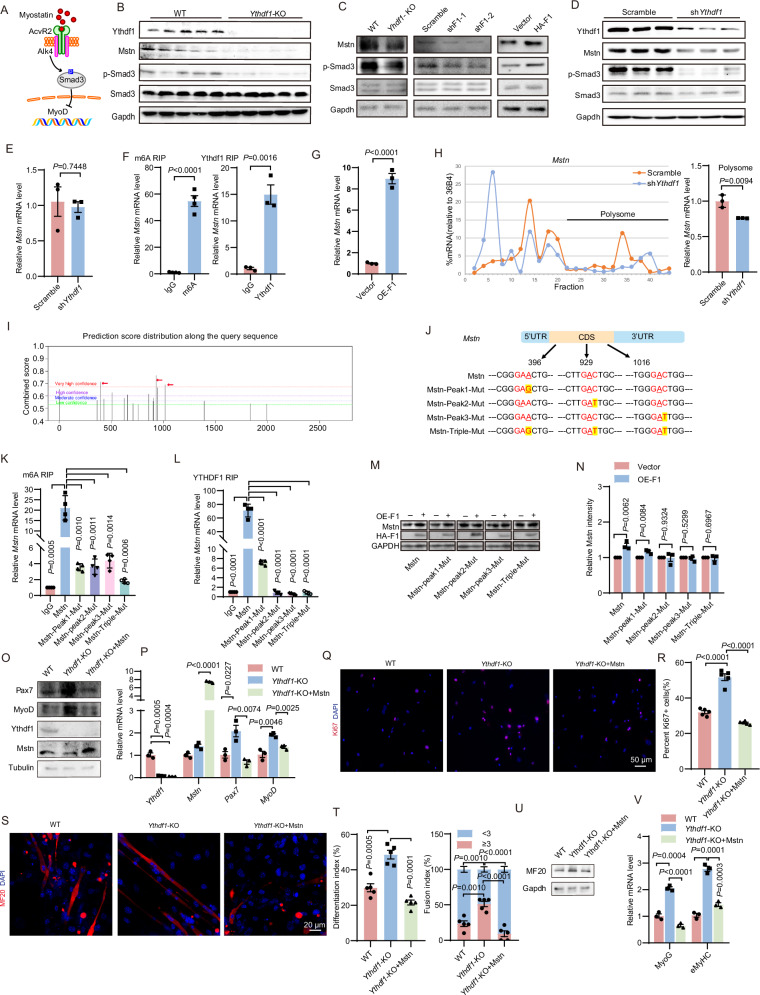
Ythdf1 promotes *Mstn* translation. **A** The myostatin signaling pathway. **B** Representative immunoblots showing Mstn, p-Smad3, and Smad3 protein levels in tibialis anterior muscles of *Ythdf1*^−/−^ and WT mice aged 4 months. **C** Representative immunoblots showing Mstn, p-Smad3, and Smad3 protein levels in WT, *Ythdf1* KO, *Ythdf1* knockdown (KD), and *Ythdf1* overexpression (OE) primary myoblasts. **D** Representative immunoblots showing the indicated protein levels in control and *Ythdf1* KD C2C12 myoblast cells. **E** qPCR analysis showing *Mstn* mRNA level in control and *Ythdf1* KD primary myoblasts. **F** m6A and Ythdf1 RIP-qPCR analysis of *Mstn* mRNA in WT myoblasts. **G** Ribosome profiling of *Mstn* mRNA in control and *Ythdf1* OE myoblasts. **H** Polysome profiling of *Mstn* mRNA in control and *Ythdf1* KD myoblasts. **I** Predicted potential m6A modification sites of *Mstn* mRNA by SRAMP. Red arrows indicate sites predicted with very high confidence. **J** Schematic diagram of the predicted m6A positions and strategies for mutation of the *Mstn* coding sequence. m6A (**K**) and YTHDF1 (**L**) RIP-qPCR analysis of *Mstn* mRNA in HEK293T cells stably overexpressing *Mstn*-WT and various *Mstn* mutants. Representative immunoblots (**M**) and quantification (**N**) showing protein levels of FLAG-*Mstn* and FLAG-*Mstn*-mutants in HEK293T cells transfected with empty vector or HA-*Ythdf1*. **O** Representative immunoblots showing protein levels of Ythdf1, Pax7, and MyoD in WT, *Ythdf1* KO, and Mstn overexpressed *Ythdf1* KO primary myoblasts. **P** qRT-PCR analysis of *Ythdf1*, *Mstn*, *Pax7*, and *MyoD* mRNA levels in WT, *Ythdf1* KO, and Mstn overexpressed *Ythdf1* KO primary myoblasts. **Q** Immunofluorescence staining of Ki67 in WT, *Ythdf1* KO, and Mstn overexpressed *Ythdf1* KO primary myoblasts. Scale bar = 20 μm. **R** Quantification of Ki67^+^ cells in (**Q**). **S** Immunofluorescence staining of MF20 in differentiated in WT, *Ythdf1* KO, and Mstn overexpressed *Ythdf1* KO primary myoblasts. Scale bar = 20 μm. **T** Quantification of differentiation index and fusion index in (**S**). **U** Representative immunoblots showing protein levels of MF20 in differentiated WT, *Ythdf1* KO, and Mstn overexpressed *Ythdf1* KO primary myoblasts. **V** qRT-PCR analysis mRNA levels of *MyoG* and *eMyHC* in differentiated WT, *Ythdf1* KO, and Mstn overexpressed *Ythdf1* KO primary myoblasts. Data represent the means ± SEM. *P* values were calculated by two-tailed unpaired Student’s *t*-test.

Mstn signaling controls skeletal muscle homeostasis and is inhibited by m6A-mediated decay of *Acvr2a* and *Asb2* mRNAs [29, 30] (Fig. 4A). To test whether Ythdf1 regulates SCs via the Mstn pathway, we first analyzed the expression levels of Mstn and p-Smad3 in the tibialis anterior muscles of *Ythdf1*^−/−^ mice. Compared to WT mice, *Ythdf1*^−/−^ mice exhibited significantly reduced Mstn and p-Smad3 protein levels in TA muscles (Fig. 4B). We further validate this finding in primary myoblasts. Interestingly, little differences in the expression of Acvr2a and Asb2 between *Ythdf1*^−/−^ and WT primary myoblasts were observed at either the protein or mRNA levels (Fig. S3A, B), suggesting that Ythdf1 does not regulate the translation or decay of these genes. Instead, a consistent downregulation of Mstn and p-Smad3 protein levels was observed in *Ythdf1*^−/−^ primary myoblasts compared with WT (Fig. 4C). In accordance with these findings, *Ythdf1* KD suppressed the protein levels of Mstn and p-Smad3, whereas *Ythdf1* OE enhanced their levels. Downregulation of Mstn and p-Smad3 was also observed in C2C12 myoblast cells with *Ythdf1* KD (Fig. 4D). Of note, *Mstn* mRNA level and stability were not affected by *Ythdf1* deficiency (Figs. 4E and S3C).

YTHDF1 enhances mRNA translation [33]. Therefore, we examined whether Ythdf1 is a bona fide m6A reader of *Mstn* mRNA. RNA immunoprecipitation using anti-m6A (m6A RIP) and anti-Ythdf1 antibodies indicated the presence of m6A modifications and the occupancy by Ythdf1 on the *Mstn* mRNA (Fig. 4F, *P* < 0.002). Interestingly, though m6A modifications on *Pax7*, *MyoD*, or *MyoG* mRNAs were detected by m6A RIP (Fig. S3D, *P* < 0.01), Ythdf1 did not bind directly to these mRNAs (Fig. S3E), nor did loss of *Ythdf1* affect their decay (Fig. S3F–H). Further ribosome profiling suggested that *Ythdf1* OE enhances *Mstn* association (Fig. 4G). In accordance with this, *Ythdf1* KD suppressed polysome association of *Mstn* mRNAs in myoblasts (Fig. 4H, *P* = 0.0094). We then investigated whether Ythdf1-mediated Mstn translation was m6A-dependent. We first used the online web tool SRAMP (Sequence-based RNA adenosine methylation site predictor) to predict potential m6A modification sites on the *Mstn* RNA sequence. Three predicted modification sites (Peak1, Peak2, and Peak3) were selected for further evaluation (Fig. 4I). FLAG-Mstn-WT and FLAG-Mstn m6A individual mutant of Peak1, Peak2, Peak3 (-Peak1/2/3 Mut) and a triple mutant (-Triple Mut) were constructed (Fig. 4J). These mutations led to a significant reduction in the m6A level of *Mstn* mRNA (Fig. 4K, *P* < 0.002). Consequently, we observed a decreased binding between YTHDF1 and *Mstn* mRNA (Fig. 4L, *P* < 0.0001). Moreover, the Peak2 and Peak3 mutants and the triple mutants totally abolished the effect of Ythdf1 in promoting *Mstn* mRNA translation (Fig. 4M, N). These data suggest that Ythdf1 recognizes m6A-modified *Mstn* mRNA to promote its translation.

Furthermore, we overexpressed *Mstn* in primary myoblasts isolated from *Ythdf1*^−/−^ mice. Markedly, the expression levels of *Pax7* and *MyoD*, and Ki67 positive staining, were all downregulated after *Mstn* OE (Fig. 4O–R, *P* < 0.05). A significant decrease in the number of MF20^+^ multinucleated myotubes was also observed following *Mstn* OE, as determined by fusion index analysis (Fig. 4S–U, *P* < 0.002). The expression levels of *MyoG* and *eMyHC* were also significantly downregulated (Fig. 4V, *P* < 0.0005). These data suggest the effect of Ythdf1 on SCs is primarily mediated by Mstn. Notably, a decrease in Mstn protein levels was observed in mice after exercise (Fig. S3I).

### *Ythdf1* deficiency promotes skeletal muscle regeneration

**Fig. 5:**
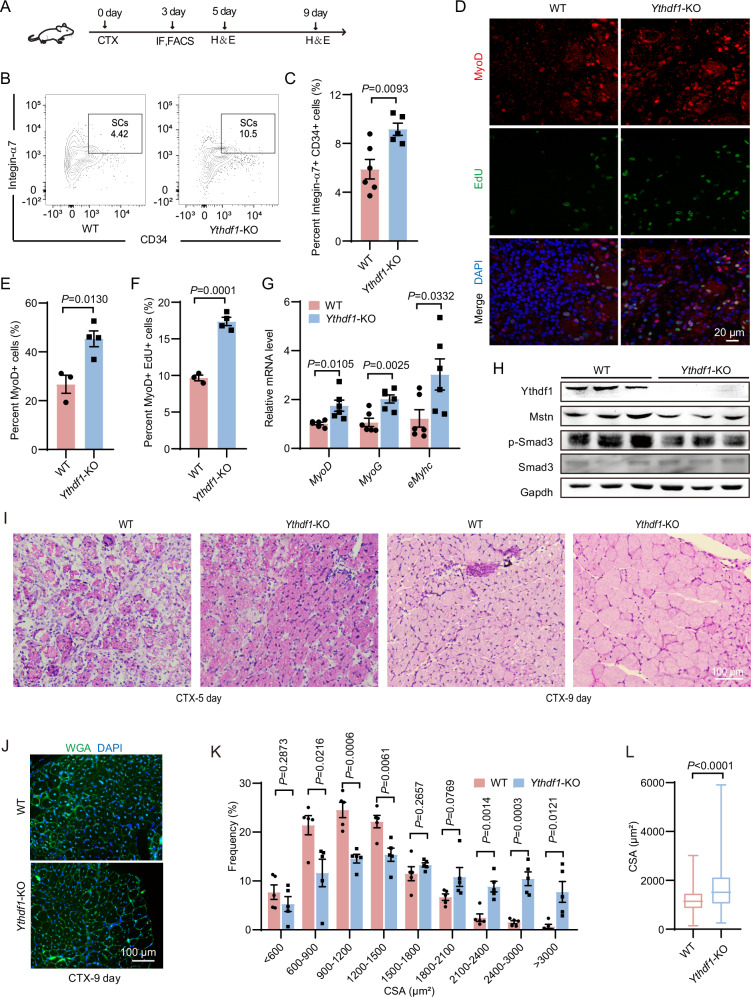
Loss of *Ythdf1* promotes muscle regeneration. **A** Schematic diagram of the mouse model of cardiotoxin (CTX)-induced skeletal muscle regeneration and sample collection. **B** Flow cytometric analysis of the SC population in muscles from *Ythdf1*^−/−^ and WT mice at 3 days after CTX injection. **C** Quantification of SCs in (**B**) (*n* = 5/6). **D** Immunofluorescent staining showing EdU and MyoD in tibialis anterior muscles from *Ythdf1*^−/−^ and WT mice 3 days after CTX injection (*n* = 3/4). **E**, **F** Quantification of EdU^+^ and MyoD^+^ cells in (**D**). **G** qPCR analysis of mRNA levels of the indicated genes in tibialis anterior muscles from *Ythdf1*^−/−^ and WT mice 3 days after CTX injection (*n* = 6). **H** Representative immunoblots showing indicated protein levels in tibialis anterior muscles from *Ythdf1*^−/−^ and WT mice at 3 days after CTX injection. **I** Representative H&E staining of tibialis anterior muscle sections from *Ythdf1*^−/−^ and WT mice 5 and 9 days after CTX injection. **J** Representative images of WGA staining of the tibialis anterior muscles from *Ythdf1*^−/−^ and WT mice 9 days after CTX injection. Percentage distribution of myofiber cross-sectional area (CSA) (**K**) and the mean CSA (**L**) in (**J**). Data represent the means ± SEM. *P* values were calculated by two-tailed unpaired Student’s *t*-test.

We next studied whether loss of *Ythdf1* affects skeletal muscle regeneration using a cardiotoxin (CTX)-induced tibialis anterior muscle injury model (Fig. 5A). We first investigated SC numbers after muscle injury. Three days after CTX injection, *Ythdf1*^−/−^ mice showed a significant increase in SC numbers compared with control mice (Fig. 5B, C, *P* < 0.01). IF staining showed that *Ythdf1* deletion significantly increased the number of MyoD^+^ cells in the tibialis anterior muscles (Fig. 5D, E, *P* = 0.013). Further EdU assays confirmed significantly increased SC proliferation in *Ythdf1*^−/−^ muscles compared to WT (Fig. 5F, *P* = 0.0001). Consistently, mRNA levels of *MyoD*, *MyoG*, and *eMyHC* were significantly elevated in tibialis anterior muscles of *Ythdf1*^−/−^ mice (Fig. 5G, *P* < 0.05). In addition, the Mstn protein level was downregulated in the tibialis anterior muscles of *Ythdf1*^*−/−*^ mice compared with controls (Fig. 5H). Furthermore, muscle regeneration was evaluated by hematoxylin and eosin (H&E) staining at 5 and 9 days after CTX treatment. Notably, regenerating fibers with centrally localized nuclei were already present in *Ythdf1*^−/−^ mice 5 days post-CTX injection, but not in WT mice (Fig. 5I). At 9 days after CTX injection, WGA staining showed enlarged myofibers in *Ythdf1*^*−/−*^ tibialis anterior muscles compared with controls (Fig. 5J–L, *P* < 0.0001 in L). These data suggested that *Ythdf1* deletion enhances acute injury-induced muscle regeneration.

### SC numbers decline with age in *Ythdf1*^−/−^ mice

**Fig. 6:**
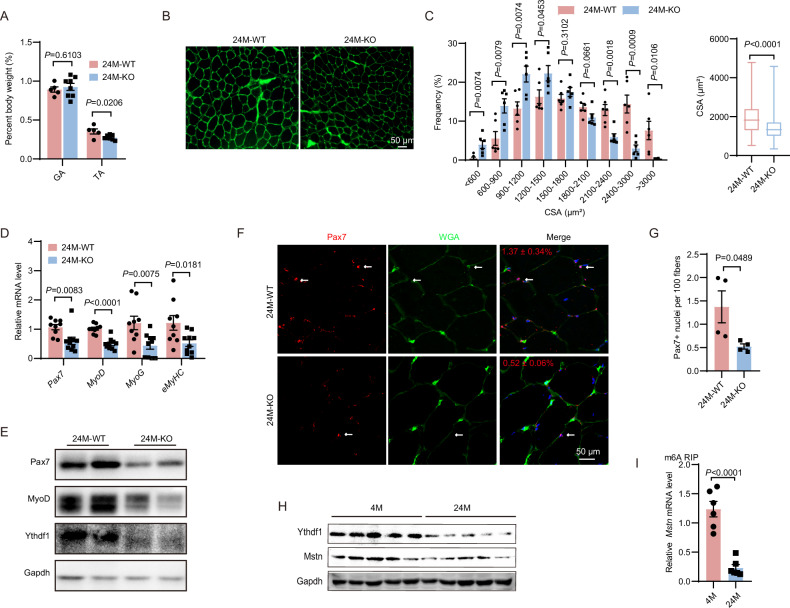
SCs decline with aging in *Ythdf1*^−/−^ mice. **A** Gastrocnemius (GA) and tibialis anterior (TA) muscle weight in 24-month-old *Ythdf1*^**−/−**^ and WT mice (*n* = 5/8). **B** Representative WGA staining of the tibialis anterior muscles in 24-month-old *Ythdf1*^**−/−**^ and WT mice. Scale bar = 50 μm. **C** Quantification of muscle fiber number and size (CSA) in (**B**) (*n* = 6). **D** qPCR analysis of expression of the indicated genes in tibialis anterior muscles of 24-month-old *Ythdf1*^**−/−**^ and WT mice (*n* = 9/10). **E** Representative immunoblots showing levels of Pax7, MyoD, and Ythdf1 proteins in 24-month-old *Ythdf1*^**−/−**^ and WT mice. **F** Immunofluorescent staining showing Pax7 in gastrocnemius muscles from 24-month-old *Ythdf1*^**−/−**^ and WT mice. **G** Quantification of muscle Pax7^+^ cells in (**F**) (*n* = 4). **H** Representative immunoblots showing proteins levels of Ythdf1 and Mstn in tibialis anterior muscles from 4-month-old and 24-month-old WT mice. **I** m6A RIP-qPCR analysis of *Mstn* mRNA in gastrocnemius muscles from 4-month-old and 24-month-old WT mice (*n* = 6). Data represent the means ± SEM. *P* values were calculated by two-tailed unpaired Student’s *t*-test.

Age-related decline in number and activation of SCs compromises muscle regenerative capacity [3]. Therefore, we investigated the sustainability of hyperactivation of SCs and enhanced muscle regeneration induced by *Ythdf1* deficiency during aging. Strikingly, we found that the weight of tibialis anterior muscles but not gastrocnemius muscles was significantly lower in *Ythdf1*^−/−^ mice compared to WT (Fig. 6A, *P* = 0.0206). In accordance with this finding, WGA staining and CSA analysis revealed that the tibialis anterior muscles of *Ythdf1*^−/−^ mice were smaller (Fig. 6B, C, *P* < 0.0001). Subsequent analysis of myogenesis revealed that mRNA levels of *Pax7*, *MyoD*, *MyoG*, and *eMyHC* were significantly downregulated in muscles of *Ythdf1*^−/−^ mice (Fig. 6D, *P* < 0.02). A corresponding reduction in Pax7 and MyoD protein levels was noticed in *Ythdf1*^−/−^ mice compared with WT at the age of 24 months, as determined by western blotting (Fig. 6E). Moreover, IF staining revealed a dramatic reduction in the number of Pax7^+^ cells in *Ythdf1*^−/−^ mice aged 24 months (Fig. 6F, G, *P* < 0.05). These data were indicative of accelerated depletion of *Ythdf1*-deficient SCs with aging. Of note, the muscle weight, myofiber size, and number of SCs all declined in aged mice compared to young mice (Fig. 2A, C–E, J, K), suggested aging-associated exhaustion of SCs and muscle atrophy. Interestingly, both Ythdf1 and Mstn protein levels were decreased in tibialis anterior muscles of aged mice compared to young (4-month-old) controls (Fig. 6H). A significant reduction in m6A modification on Mstn mRNA in tibialis anterior muscles was revealed by MeRIP assay in aged mice (Fig. 6I, *P* < 0.0001). Together, the data indicate accelerated depletion of *Ythdf1*-deficient SCs with aging, and the decline of the Ythdf1-Mstn axis in skeletal muscles is also obvious in normal aging.

## Discussion

Here, we elucidated the role of RNA m6A modification in skeletal muscle remodeling by endurance exercise training. Mechanistically, Ythdf1 recognizes the m6A-modified *Mstn* mRNA to promote its translation and downregulation of Mstn hyperactivates SCs, thus promoting skeletal muscle growth and injury-induced regeneration. In response to long-term endurance exercise, skeletal muscle boosts mitochondria biogenesis to adapt the increase of energy demand [34]. Consistently, deletion of *Ythdf1* increased oxidative metabolism in skeletal muscle and enhanced endurance performance. Interestingly, endurance exercise training also suppressed the expression of *Mettl3/14*, and the overall m6A level was decreased. It is plausible to speculate that downregulation of *Mettl3/14* may contribute to the low level of Mstn and thus skeletal muscle remodeling. In future, it is worthwhile to study how exercise suppresses *Ythdf1* and *Mettl3/14* transcript levels.

Previous studies have shown that mechanical overload induces muscle hypertrophy via the Acvr2a-Smad-MyoD signaling [29, 30]. Mettl3 catalyzes m6A-modified *Acvr2a*, inducing Ythdf2-mediated RNA decay and therefore skeletal muscle hypertrophy [29]. Ythdf2 also enhances the decay of ubiquitin ligase *Asb2* mRNA, further inhibiting the Acvr2a-Smad-MyoD signaling [30]. In this study, we identified an additional regulation of skeletal muscle remodeling though the Mstn-Acvr2a-Smad-MyoD signaling cascade. Overall, m6A modifications on various signaling pathway elements and the differential roles of YTHDFs in regulating RNA fate ensures a cascading constraint of the Mstn-Acvr2a-Smad-MyoD signaling from over-activation or over-inhibition, thus preventing skeletal muscle overgrowth or atrophy, respectively. Our data provide crucial insights into the mechanism by which complex muscle fate determination is finely orchestrated by YTHDFs reading different transcripts in the same pathway. How such specificity is achieved merits further investigation.

We found that *Ythdf1* deficiency increased proliferation and differentiation of in vitro cultured primary myoblasts, indicating the regulation of SCs by Ythdf1 at least partially is cell autonomous. Supporting this notion, muscle regeneration of *Ythdf1* KO mice was enhanced in CTX-induced muscle injury model. However, the local and systemic environment can affect skeletal muscle regeneration by regulating SCs function, such as macrophage, regulatory T cells, neuromuscular junctions, and endothelial cells [3, 35]. A MuSC-specific *Ythdf1* KO mice model may help elucidate the role of SCs in muscle phenotypes observed in *Ythdf1*-deficient mice. Both muscle fiber number and muscle fiber size account for the increase of muscle mass in *Mstn*^−/−^ mice [7]. The muscle hypertrophy in *Ythdf1* KO mic could also be resulted from increased protein translation and/or reduced protein degradation of muscle fibers [15–19].

The maintenance of the SCs pool is crucial to prevent aging-related muscle decline [31, 32]. SCs can be activated by exercise, accompanied by an increase in the transcription of genes encoding myogenic regulators [25, 26]. Interestingly, SCs were hyperactivated in young *Ythdf1*^−/−^ mice but exhausted in old mice. In these mice, constitutive inhibition of Mstn signaling may disrupt the balance between self-renewal and activation of SCs. In accordance with our findings, long-term activation of SCs led to a depleted SC pool and impaired muscle regeneration in old mice [36, 37].

In summary, we discovered that loss of *Ythdf1* has beneficial effects on muscle growth and physical performance but is detrimental to long-term skeletal muscle homeostasis. The Ythdf1-dependent regulation of Mstn signaling extends our understanding of the complexity of m6A-mediated skeletal muscle remodeling.

## Methods

### Animal studies

We commissioned Cyagen Biosciences (Suzhou) Biotech Co., Ltd. (China) to construct an *Ythdf1* gene knockout allele on a C57BL/6N mouse background via the CRISPR-Cas9 system. All mice were maintained at 22 °C with 12-h light/dark cycles unless otherwise described.

For exercise training, 10-week-old male mice were trained 5 days a week for 4 weeks using a motorized treadmill [38, 39]. Prior to training, mice were pre-adapted to the treadmill (15° incline) for 15 min per day for 3 days at a gradually increased speed (10–17 m/min). Daily exercise training was achieved with 30 min of forced running on a rodent treadmill at a speed of 17 m/min.

Running endurance test was conducted as described previously [40]. Ten-week-old male mice were acclimated to the treadmill (15° incline) at 10 m/min for 5 min per day for 2 days. Running performance were tested with a gradually increasing speed from 10 m/min to 25 m/min till mice failed. Exhaustion was defined as mice were sustained receiving >20 shock within 10 s at 0.5 mA stimulation.

For muscle injury and regeneration, adult mice were anesthetized using an isoflurane inhalation anesthesia system (F500 Series, EZVET, China). Tibialis anterior muscle injury was induced in adult mice by injection of 50 μl cardiotoxin (CTX; 5 µg solution, Boyao, Shanghai, China). At specified time points post-injury, mice were euthanized by CO_2_ asphyxiation, and tibialis anterior muscles were harvested to evaluate the progress of regeneration and repair.

### Western blotting and antibodies

Total proteins were extracted from cells or homogenized tissue samples lysed using ice-cold RIPA buffer (P0013B, Beyotime, Shanghai, China) supplemented with 1× protease inhibitor cocktail and phosphatase inhibitor. The protein concentration was determined using the BCA Protein Assay Kit (23227, Thermo Fisher, Massachusetts, USA). Equal amounts of protein extracts were subsequently separated by sodium dodecyl sulfate-polyacrylamide gel electrophoresis (SDS-PAGE) and transferred to a polyvinylidene difluoride membrane (IPVH00010, Millipore, Germany). Subsequently, the membranes were blocked with 5% bovine serum albumin (BSA) for 1 h at room temperature before incubation with antibodies overnight at 4 °C for detection of the following: p-Smad3 (9520s, Cell Signaling Technology, Massachusetts, USA), MyoD (PA5-23078, Thermo Fisher), Pax7 (307932, DSHB, Iowa, USA), Mstn (19142-1-AP, Proteintech, Wuhan, China), Ythdf1 (ab252346, Abcam, Cambridge, UK), Ki67 (ab15580, Abcam) and Gapdh (60004-1-IG, Proteintech). The membranes were then incubated with corresponding secondary antibodies for 1 h at room temperature. Immunoreactivities were determined using the enhanced chemiluminescence (ECL) method (34578, Thermo Fisher). The grayscale value of each protein band was quantified using ImageJ software (V1.52).

### Primary myoblast isolation, culture, and differentiation

SC-derived primary myoblasts were isolated from the gastrocnemius and tibialis anterior skeletal muscles of *Ythdf1*^−/−^ and WT mice aged 6–8 weeks. The muscles were minced and digested by digestion medium containing 2.9 U/ml type II collagenase (17101015, Gibco, Thermo Fisher) and 2.4 U/ml dispase (9068-59-1, Shifeng, China) in phosphate-buffered saline (PBS) with 2.5 mM CaCl_2_ for 60 min at 37 °C. Each mouse muscle sample was digested in 3 ml of digestion medium. The digestions were terminated by the addition of 4 ml F-10 Ham’s medium (11550043, Gibco, Thermo Fisher) containing 20% FBS (Pan Seratech, Germany). Cells were then filtered through a 70 µm sterilized cell strainer from debris, centrifuged at 2000 × *g* for 5 min, and cultured in growth medium (F-10 Ham’s medium supplemented with 20% FBS, 2.5 ng/ml basic fibroblast growth factor (FGF), and 1% penicillin-streptomycin) on collagen-coated cell culture plates at 37 °C under 5% CO_2_. For differentiation, primary myoblasts were grown in differentiation medium (DMEM supplemented with 2% horse serum and 1% penicillin-streptomycin) for 3 days.

### RNA immunoprecipitation

Cells are first washed with pre-cooled PBS and then crosslinked using a UV crosslinker (SZ03, Shanghai Jingxin, China). Cells were then lysed in lysis buffer (50 mM HEPES (pH 7.5), 150 mM KCl, 2 mM EDTA, 0.5% NP-40) containing DTT, protease inhibitors and RNase inhibitor followed by centrifugation at 14,000 × *g* for 15 min. 10% of supernatant was collected as the input. The remaining supernatant was incubated with 5 μg of anti-YTHDF1 (17479-AP, Proteintech) antibody, or IgG-conjugated protein A/G agarose beads at 4 °C overnight. Samples were washed three times with washing buffer (50 mM HEPES (pH 7.5), 300 mM KCl, 0.05% NP-40, and 0.5 mM DTT) containing protease inhibitors and RNase inhibitor, subjected to DNase I (2 U/ml) digest, and eluted in elution buffer (50 mM Tris-HCl (pH 7.5), 150 mM NaCl, 0.05% NP-40, 1 mM MgCl_2_) containing 1 μl SDS (10%) and 5 μl proteinase K. RNA was extracted using the TRIzol reagent RNAiso Plus (9109, Takara, Japan) for subsequent real-time quantitative PCR analysis.

### RNA extraction, qPCR, and m6A dot blot

Total RNA from cells or tissues was extracted using TRIzol reagent RNAiso Plus (9109, Takara, Japan) according to the manufacturer’s instructions. For cDNA synthesis, 2 μg of total RNA was used in a 20 μl reaction volume, and reverse transcription was performed using Evo M-MLV RT Master Mix (AG11706, Accurate Biology, China) following the manufacturer’s protocol. Quantitative PCR (qPCR) was then conducted using SYBR Green qPCR Master Mix (11201, Yeasen, China) on a CFX Connect Real-Time System (Bio-Rad). GAPDH was used as an endogenous control.

For m6A dot blot analysis, 300 ng of total RNA sample was added to an Amersham Hybond-N^+^ membrane (INYC00010, Biosharp, China). Following UV cross-linking, the membrane was washed with TBST buffer, blocked with 5% skim milk in TBST for 1 h at room temperature, and then incubated overnight at 4 °C with an anti-m6A antibody (1:1000, Synaptic Systems, Germany). Subsequently, the membrane was incubated with the corresponding HRP-conjugated secondary antibody at room temperature for 1 h. The blots were developed using an ECL system, and densitometric analysis was performed using ImageJ software.

### Ribosome profiling

Cells were treated with 100 µg/ml cycloheximide (CHX) for 10 min and then washed twice with 5 ml cold PBS containing CHX (100 µg/ml). After centrifugation at 1000 × *g* for 5 min, a total of 400 µl ice-cold lysis buffer, consisting of 20 mM Tris·HCl (pH 7.4), 150 mM NaCl, 5 mM MgCl_2_, 1 mM DTT, 100 µg/ml CHX, 1% (vol/vol) Triton X-100, 25 U/ml Turbo DNase I, freshly added 1:100 protease inhibitor cocktail, and 40 U/ml RNase inhibitor was added to the cell pellet. The cells were lysed on ice for 15 min and homogenized 10 times using a 26-G needle. The lysate was clarified by centrifugation at 14,000 × *g* for 10 min at 4 °C, and the soluble supernatant was collected. Subsequently, 7.5 µl RNase I (100 U/µl) was added to 300 µl of lysate and incubated at room temperature for 45 min. The reaction was stopped by adding 10 µl RNase inhibitor. The processed sample was transferred into the centrifuge tube. A 1 M sucrose solution (1 M sucrose, 20 mM Tris·HCl (pH 7.4), 150 mM NaCl, 5 mM MgCl_2_, 1 mM DTT, 100 µg/ml CHX, and 40 U/ml RNase inhibitor) was slowly added to the bottom of the sample to form a “sucrose cushion”. Ribosomal particles were pelleted by centrifugation at 180,000 × *g* for 4 h at 4 °C. RNA was subsequently extracted, reverse-transcribed, and analyzed by qPCR.

### Histology and immunofluorescence staining

Whole muscle tissues of WT and *Ythdf1*^*−/−*^ mice were dissected and frozen immediately in OCT compound. Frozen muscles were sectioned (10 µm thickness) using a Leica CM1850 cryostat. The sections were subjected to H&E or IF staining. For IF staining, sections, or cultured cells on coverslip were fixed in 4% paraformaldehyde in PBS for 10 min, quenched with 100 mM glycine for 10 min, and incubated in blocking buffer (3% bovine serum albumin, 0.3% Triton X-100 in PBS) for ≥1 h. After incubation with primary antibodies overnight at 4 °C, sections were washed and incubated with secondary antibodies for 2 h at room temperature, followed by a 5-min incubation with DAPI at room temperature. The following primary antibodies were used: Ki67 (ab15580, Abcam, 1:500), MF20 (14-6503-82, Thermo Fisher, 1:500), and MyoD (PA5-23078, Thermo Fisher, 1:500). The following secondary antibodies were used: Alexa Fluor 488-conjugated goat anti-mouse IgG (A11001, Thermo Fisher, 1:500), Alexa Fluor 594-conjugated donkey anti-mouse IgG (A21203, Thermo Fisher, 1:500), Alexa Fluor 488-conjugated donkey anti-rabbit IgG (A21206, Thermo Fisher, 1:500), and Alexa Fluor 594-conjugated goat anti-rabbit IgG (A11037, Thermo Fisher, 1:500). Images of WT and *Ythdf1*^*−/−*^ samples were captured with a Leica DM 6000B microscope using identical parameters.

### Flow cytometric analysis

SC isolation and enrichment were performed according to established methods [41]. Briefly, the skeletal muscle isolated from mice was digested with 2 μg/ml collagenase I, 2.4 U/ml dispase I, 10 ng/ml DNase I, 0.4 mM CaCl_2_, and 5 mM MgCl_2_ for 90–120 min at 37 °C. The cell suspension was filtered through a 70 μm nylon filter (Falcon) and mononuclear cells were collected and subjected to flow cytometric analysis (BD FACS AriaII) after immunostaining with following antibodies against the following markers: CD45 (103112, BioLegend, California, USA, 1:200), CD31 (561073, BD Bioscience, California, USA, 1:200) and Sca1 (565507, BD Bioscience, 1:200), CD11b (48-0112-82, Thermo Fisher, 1:500), CD34 (128621, BioLegend, 1:300) and integrin α7 (K0046-4, MBL, Nagoya, Japan, 1:20). Subsequently, the data were analyzed using FlowJo software, version 10.

### Statistical analysis

All data were presented as mean ± standard error (SEM), with dots indicating individual biological samples within a group. Each experiment was repeated on at least three independent occasions to ensure reproducibility. For normally distributed data, statistical analysis of differences between the two groups was performed using the unpaired two-tailed *t*-test. *P* < 0.05 was considered to indicate statistical significance. Data analysis was performed using GraphPad Prism 8 (GraphPad Software).

## Supplementary information

Supplementary Figures

Original data_qPCR

Original data_Uncropped western blots

## Data Availability

All data needed to evaluate the conclusions in the paper are present in the paper and/or the Supplementary Materials.
