# Supplementary material for: Endurance exercise remodels skeletal muscle by suppressing Ythdf1-mediated myostatin expression
Source: Cell Death Dis. 2025 Feb 13;16(1):96. doi: 10.1038/s41419-025-07379-5 (PMC11825732; doi:10.1038/s41419-025-07379-5)
Supplement: Supplementary file 1 — Supplementary Figures [file 41419_2025_7379_MOESM1_ESM.pdf]

Supplementary Figures

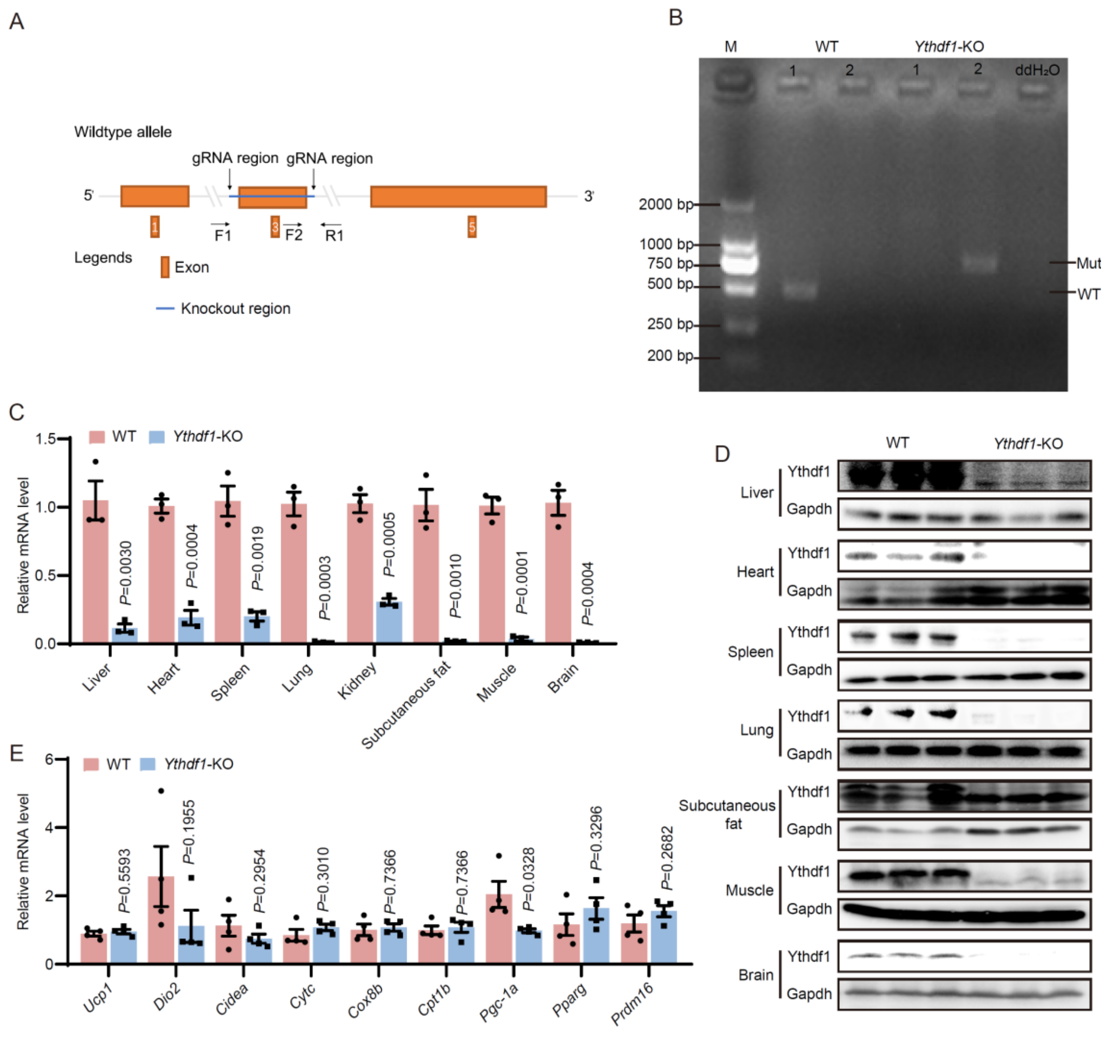

Fig. S1 Generation and characterization of *Ythdf1* knockout (KO) mice

(**A**) *Ythdf1* KO allele construction and genotyping strategy.

(**B**) PCR-based genotyping analysis of *Ythdf1*$^{-/-}$ and WT mice.

(**C**) qPCR analysis of *Ythdf1* mRNA levels in the indicated tissues of *Ythdf1*$^{-/-}$ and WT mice aged 3 months (n = 3).

(**D**) Representative immunoblots showing Ythdf1 protein levels in the indicated tissues of *Ythdf1*$^{-/-}$ and WT mice aged 3 months.

(**E**) qPCR analysis of indicated genes expression in brown adipose tissue (BAT) of *Ythdf1*$^{-/-}$ and WT mice aged 3 months (n = 4).

Data represent the means ± SEM. *P*-values were calculated by two-tailed unpaired Student's *t*-test.

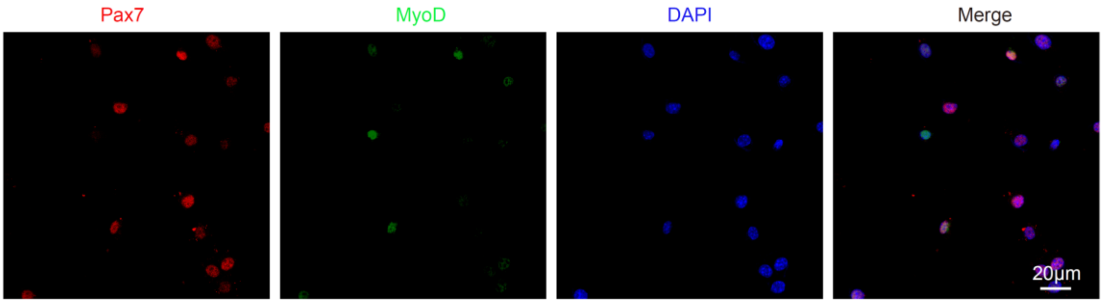

Fig. S2 Identification of primary SCs

Immunofluorescence staining of Pax7 and MyoD in WT SCs. Scale bar = 20 μm.

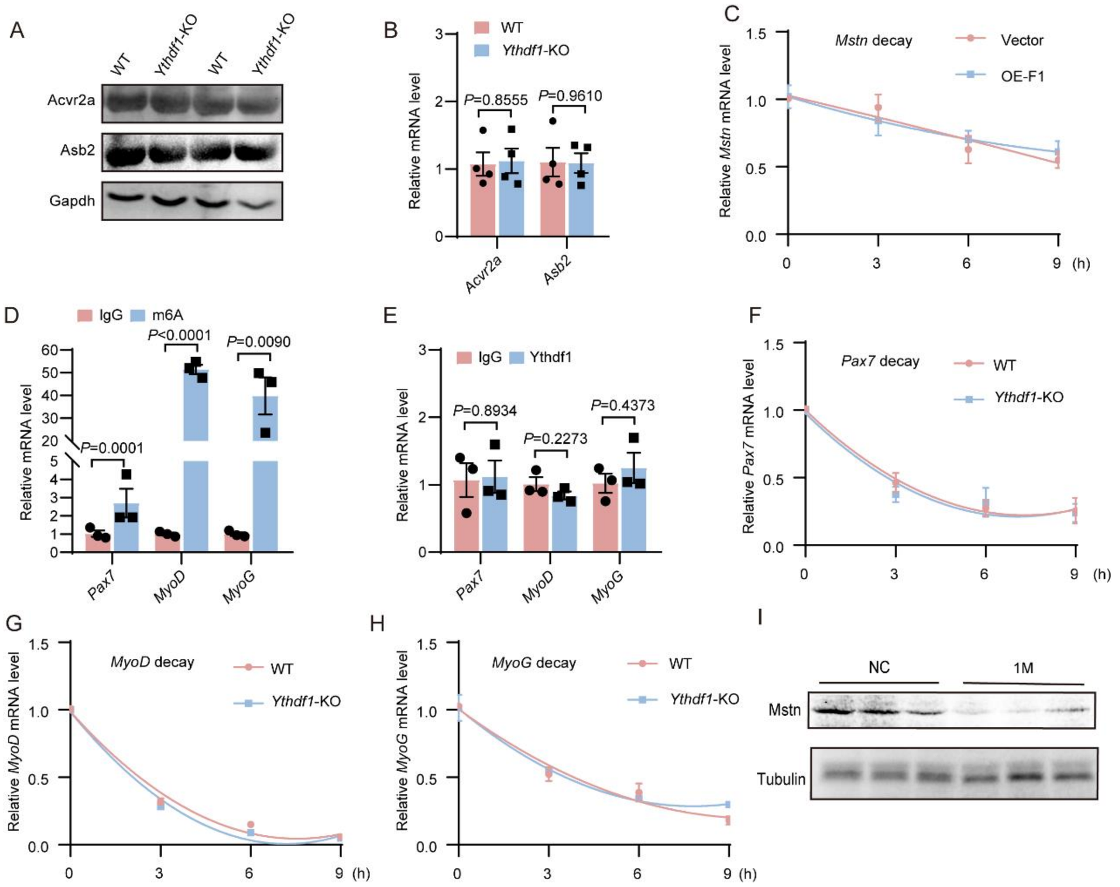

Fig. S3 Ythdf1 promotes *Mstn* translation

(**A**) Representative immunoblots showing Acvr2a and Asb2 protein levels in *Ythdf1*$^{-/-}$ and WT primary myoblasts.

(**B**) qRT-PCR analysis of mRNA levels of *Acvr2a* and *Asb2* in *Ythdf1*$^{-/-}$ and WT primary myoblasts.

(**C**) qRT-PCR analysis of mRNA levels of *Mstn* in WT and *Ythdf1* OE myoblasts at different times after actinomycin D treatment.

(**D-E**) m6A (**D**) and Ythdf1 (**E**) RIP-qPCR analysis of *Pax7, MyoD* and *MyoG* mRNA levels in *Ythdf1*$^{-/-}$ and WT primary myoblasts.

(**F-H**) qRT-PCR analysis of mRNA levels of *Pax7* (**F**)*, MyoD* (**G**) and *MyoG* (**H**) in *Ythdf1*$^{-/-}$ and WT primary myoblasts at different times after actinomycin D treatment.

(**I**) Representative immunoblots showing Mstn protein level of tibialis anterior muscles in sedentary or trained mice.

Data represent the means ± SEM. *P*-values were calculated by two-tailed unpaired Student's *t*-test.
