# Supplementary material for: Endurance exercise remodels skeletal muscle by suppressing Ythdf1-mediated myostatin expression
Source: Cell Death Dis. 2025 Feb 13;16(1):96. doi: 10.1038/s41419-025-07379-5 (PMC11825732; doi:10.1038/s41419-025-07379-5)

Figure 2G

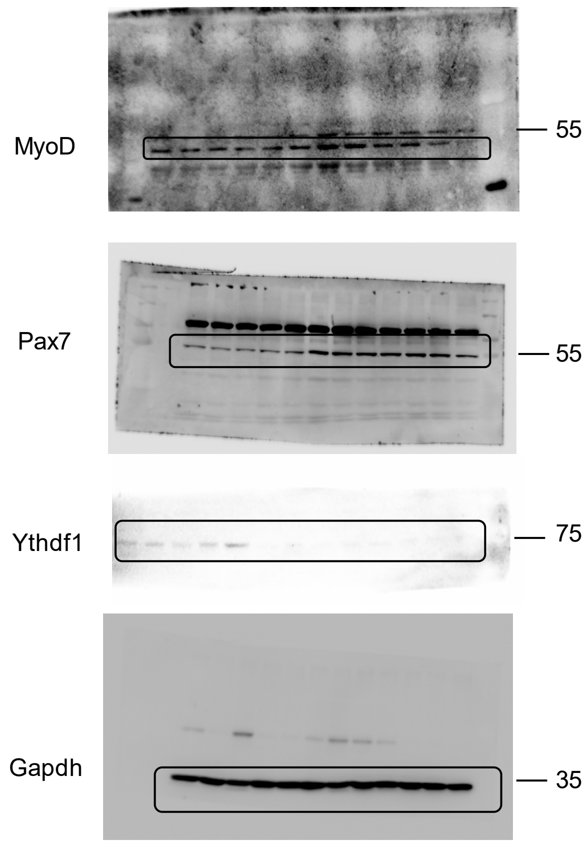

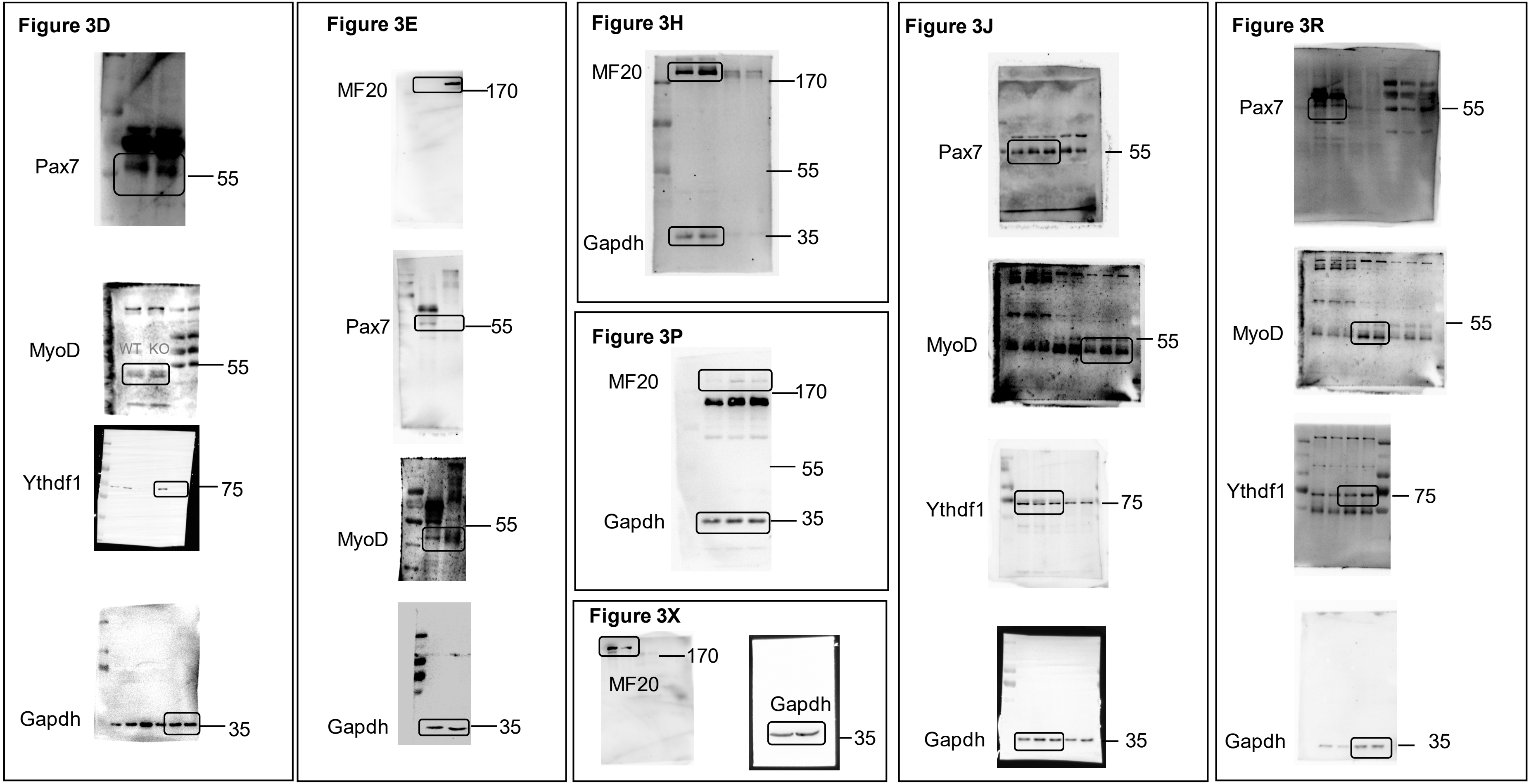
Figure 3D
Pax7
55
MyoD
WT KO
55
Ythdf1
75
Gapdh
35
Figure 3E
MF20
170
Pax7
55
MyoD
55
Gapdh
35
Figure 3H
MF20
170
55
Gapdh
35
Figure 3P
MF20
170
55
Gapdh
35
Figure 3X
170
MF20
Gapdh
35
Figure 3J
Pax7
55
MyoD
55
Ythdf1
75
Gapdh
35
Figure 3R
Pax7
55
MyoD
55
Ythdf1
75
Gapdh
35

Figure 4B

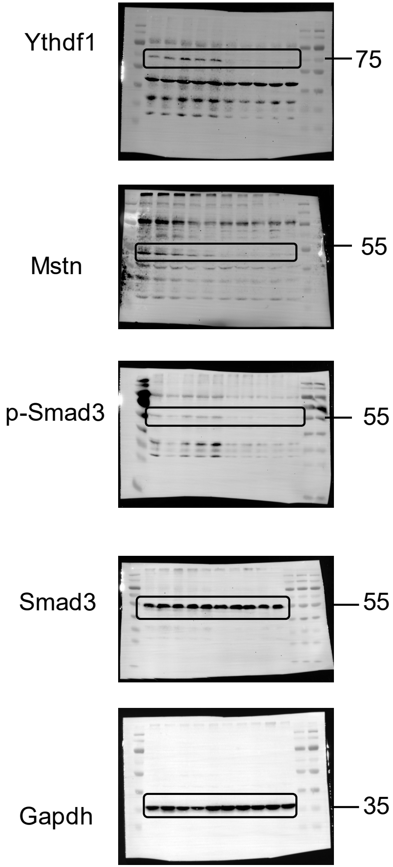

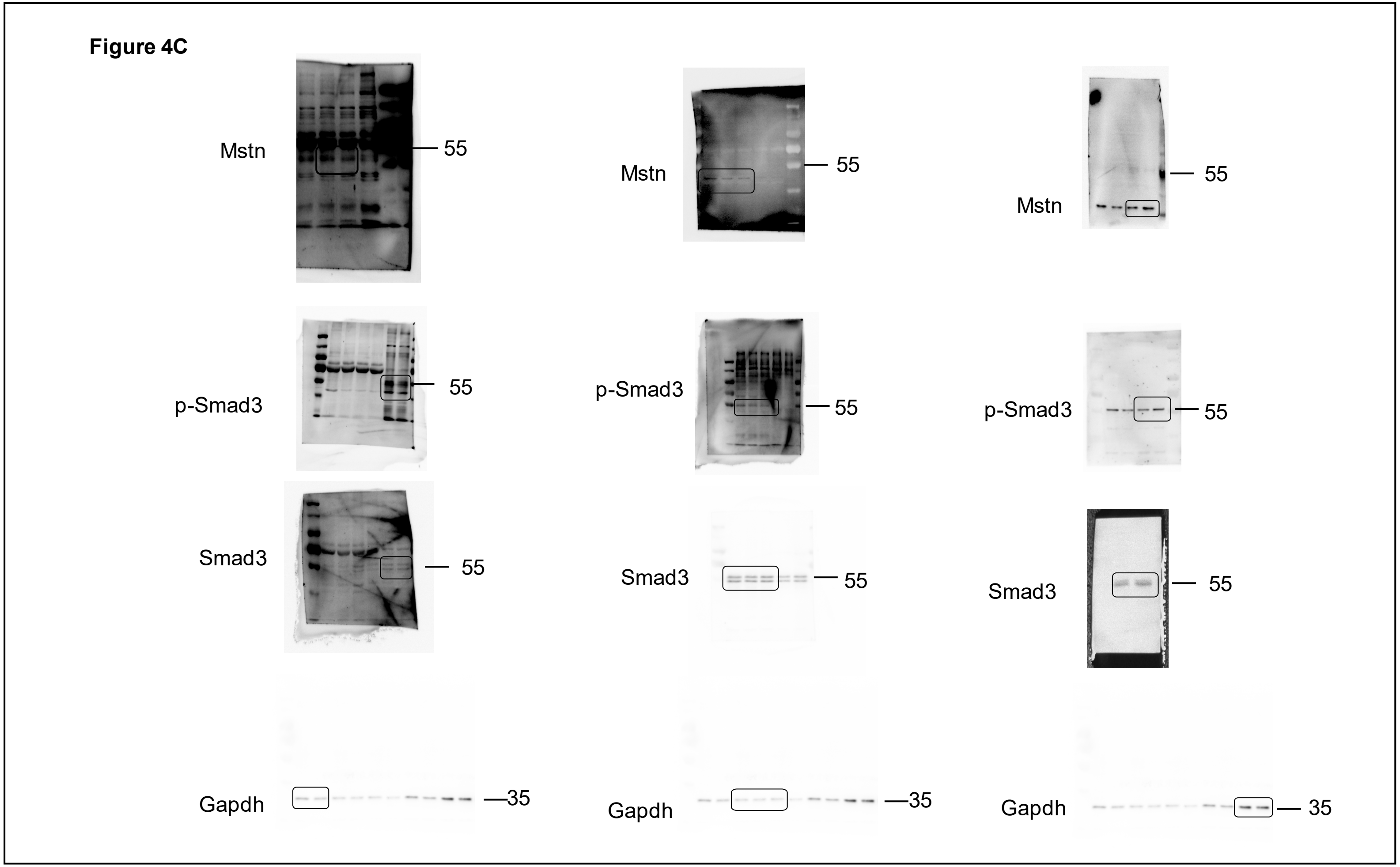
Figure 4C
Mstn
55
Mstn
55
Mstn
55
p-Smad3
55
p-Smad3
55
p-Smad3
55
Smad3
55
Smad3
55
Smad3
55
Gapdh
35
Gapdh
35
Gapdh
35

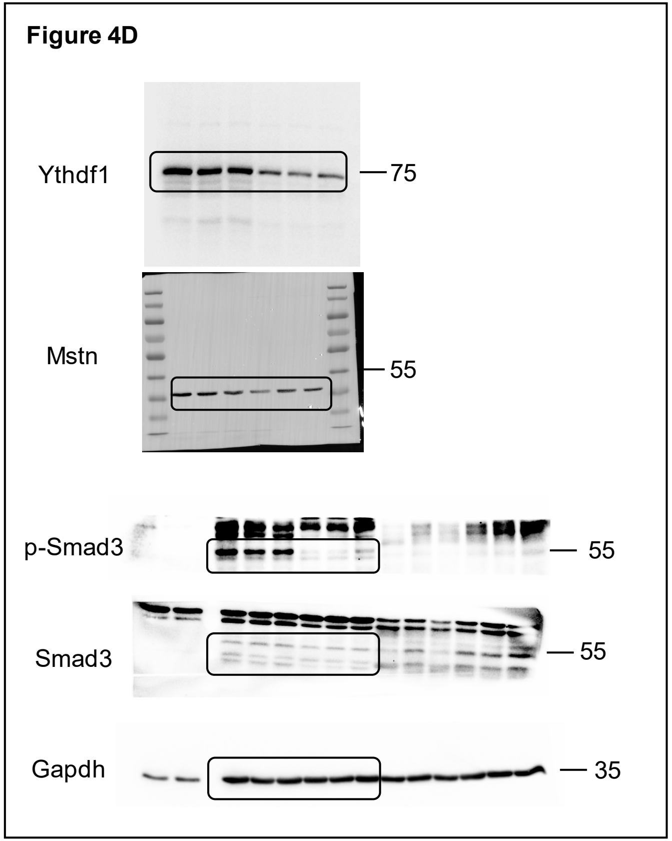
Figure 4D
Ythdf1
75
Mstn
55
p-Smad3
55
Smad3
55
Gapdh
35

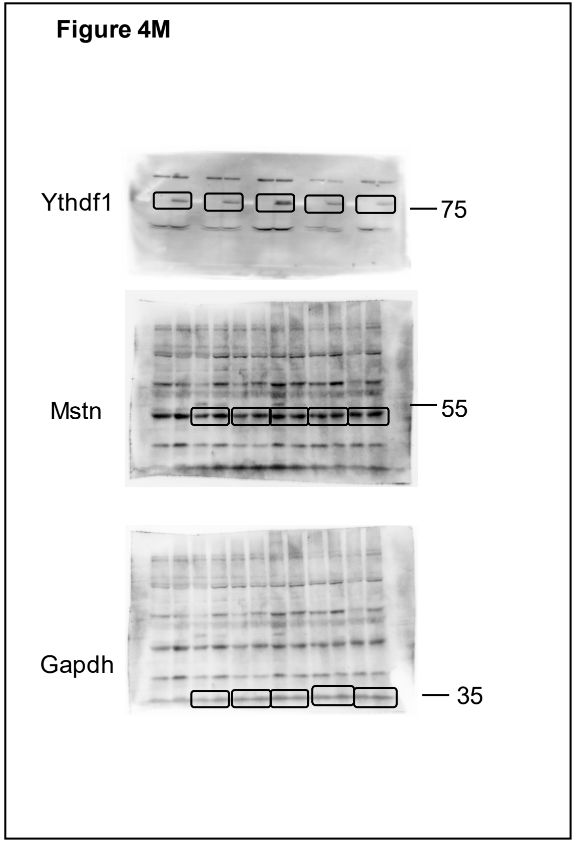
Figure 4M
Ythdf1
75
Mstn
55
Gapdh
35

Figure 4O

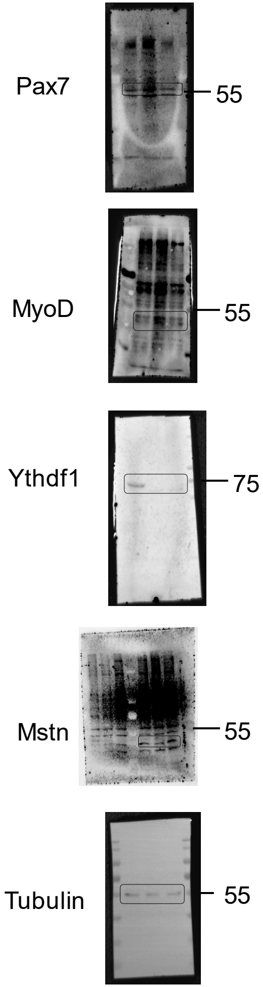

Figure 4U

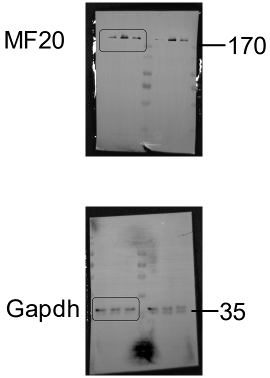

Figure 5H

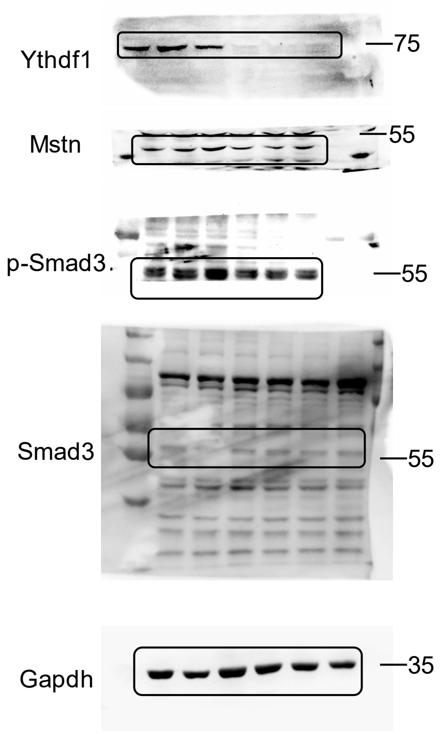

Figure 6E

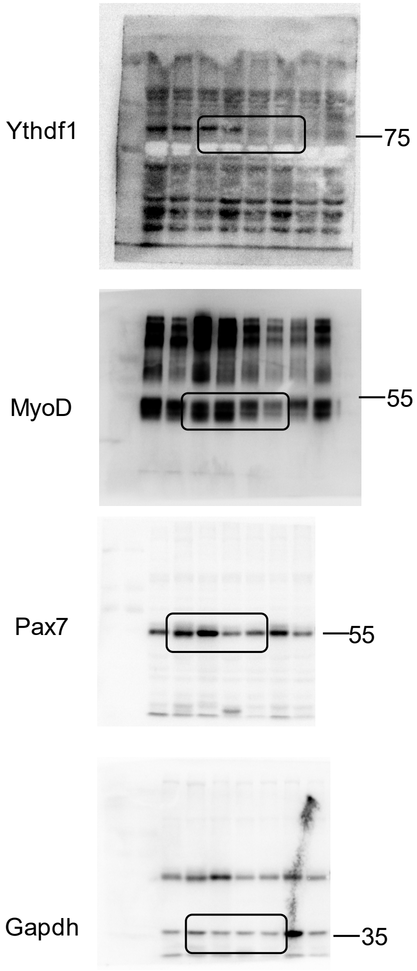

Figure 6H

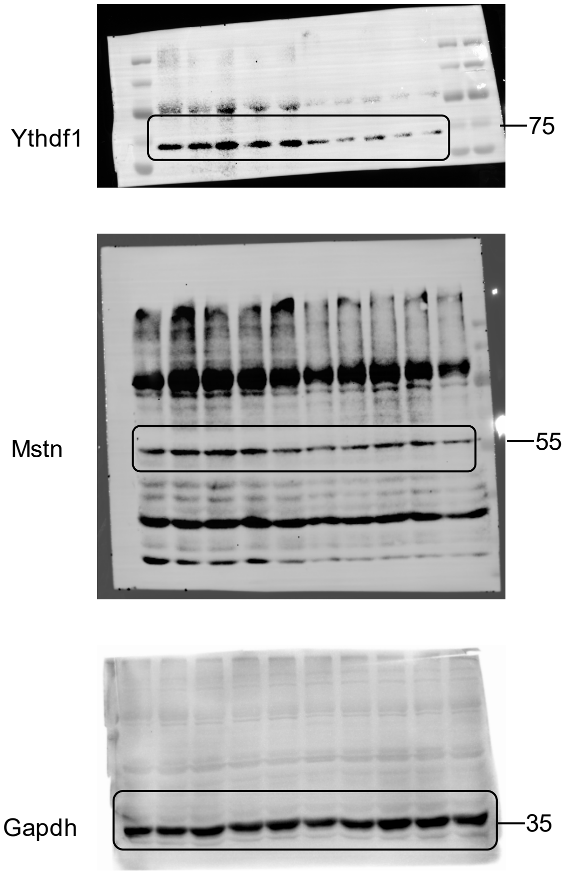

Figure S1D

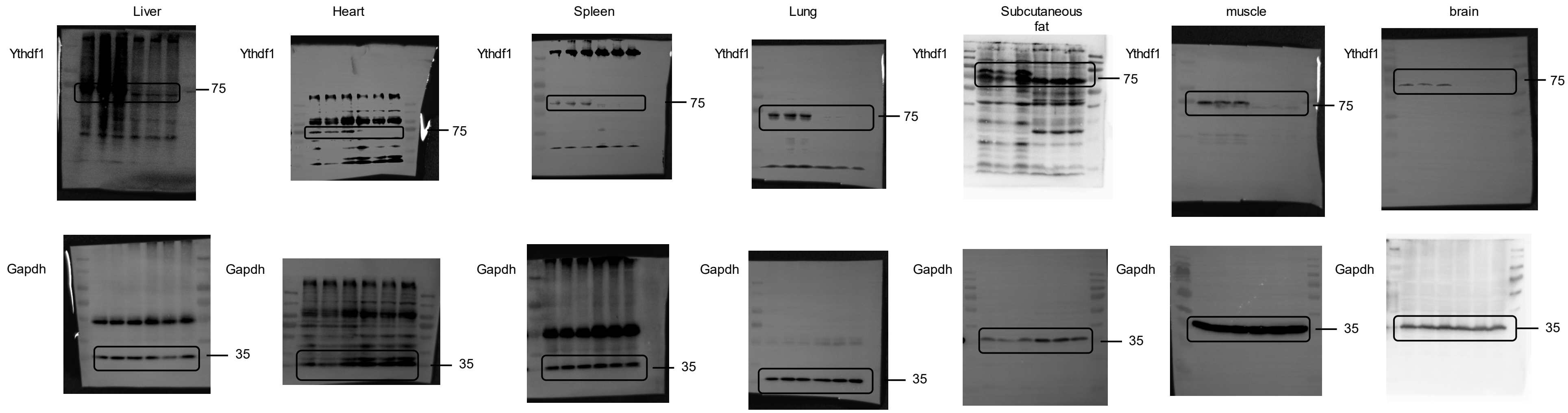
Liver
Heart
Spleen
Lung
Subcutaneous fat
muscle
brain
Ythdf1
Ythdf1
Ythdf1
Ythdf1
Ythdf1
Ythdf1
Ythdf1
75
75
75
75
75
75
75
Gapdh
Gapdh
Gapdh
Gapdh
Gapdh
Gapdh
Gapdh
35
35
35
35
35
35
35

Figure S3A

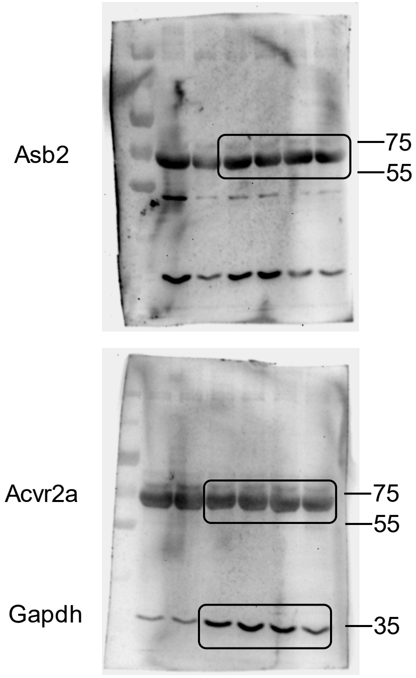

Figure S3I

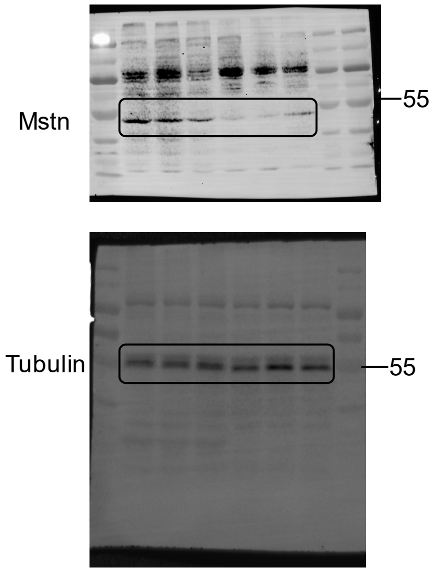

Supplement: Supplementary file 3 — Original data_Uncropped western blots [file 41419_2025_7379_MOESM3_ESM.pdf]
